# Greek Translation, Cultural Adaptation and Validation of the Mini Sarcopenia Risk Assessment Questionnaire, to Evaluate Sarcopenia in Greek Elderly at a Hospital Setting

**DOI:** 10.3390/nursrep13010037

**Published:** 2023-03-03

**Authors:** Anna Pantouvaki, Grigorios Kastanis, Evridiki Patelarou, Kalliopi Alpantaki, Christos Kleisiaris, Michail Zografakis-Sfakianakis

**Affiliations:** 1 Department of Nursing, School of Health Sciences, Hellenic Mediterranean University, 71410 Heraklion, Greece; 2 Department of Physiotherapy, General Hospital, “Venizeleio-Pananeio”, 71409 Heraklion, Greece; 3 Department of Orthopaedics, General Hospital, “Venizeleio-Pananeio”, 71409 Heraklion, Greece

**Keywords:** sarcopenia, screening tools, MSRA-7, MSRA-5, SARC-F, translation, validation

## Abstract

Sarcopenia is a progressive aging syndrome with severe socioeconomic costs. Therefore, the early diagnosis of sarcopenia is required to secure early treatment and to enhance quality of life. The Mini Sarcopenia Risk Assessment (MSRA) questionnaire, which is available in seven-item (MSRA-7) and five-item (MSRA-5) versions, is a sarcopenia screening tool that was translated into Greek, adapted, and validated in this study. The present study was developed in an outpatient hospital setting, from April 2021 to June 2022. The MSRA-7 and MSRA-5 questionnaires were translated backwards and forwards and adapted to the Greek language. To validate the MSRA questionnaire as a pre-screening tool to identify the risk of sarcopenia in the older Greek population, both the MSRA-7 and MSRA-5 versions were correlated with the Greek version of the SARC-F questionnaire, which is a widely accepted and well-known tool used in sarcopenia screening. Ninety elderly subjects aged 65–89 years-old with no mobility impairments participated in this study. The questionnaires’ content validity was assessed using the Content Validity Ratio, and the Content Validity Index was calculated for the instrument. The intra-rater reliability was assessed by calculating the Intra-class Correlation Coefficient between the initial assessment and the reassessment of the MSRA questionnaire, which was 0.986, with a 95% Confidence Interval of 0.961–0.995. Concurrent validity was assessed between the Greek MSRA questionnaires and the SARC-F questionnaire using the Spearman’s rank correlation coefficient (p). The Greek MSRA-7 questionnaire had a very high correlation with the SARC-F questionnaire (rho = −0.741, *p* < 0.001), as did the Greek MSRA-5 questionnaire (rho = −0.724, *p* < 0.001). The proofs of content validity, concurrent validity, and intra-rater reliability provided for the Greek versions of the MSRA, designated them as reliable pre-screening tools for the detection of sarcopenia in the older population and in clinical practice.

## 1. Introduction

Sarcopenia is a progressive aging syndrome, characterized by the loss of skeletal muscle mass and by the loss of muscle function, which is associated with an increased risk of falls, injuries, physical disability, lack of independence, hospitalization and even death [1]. Although it was defined as an older age-related disorder until now, it began to be present even in middle-aged subjects, resulting in serious effects upon quality of life. The global prevalence of sarcopenia and severe sarcopenia is quite lacking, and its estimation is reported in only a few systematic reviews, based on the various diagnostic criteria and definition used. However, it seems that it ranges between 0.2% and 86.5% [2]. It is also reported that sarcopenia prevalence in healthy older adults is 10% [3], while in community dwelling people, it is present in 11% of men and 9% of women [4]. The estimated prevalence of sarcopenia differs due to dissimilar population characteristics and living situations, and to the different diagnostic tools used to assess sarcopenia. The estimation of sarcopenia prevalence is also lacking in Greece. The first attempt to estimate Greek sarcopenia prevalence was made by Tsekoura et al., according to the EWGSOP2 criteria, which revealed that there is a probable sarcopenia prevalence rate of 25.4% and a sarcopenia rate of 13.2% in elderly adults, a quite important rate that urges the necessity of the early diagnosis of this muscle disorder [5].

One of the most common research topics concerning sarcopenia is its diagnosis. Several definitions have been proposed including measures such as muscle mass (assessed by imaging techniques: dual energy X-ray, absorptiometry (DXA), computerized tomography (CT), magnetic resonance imaging (MRI)), muscle strength (assessed by handgrip dynamometer) and functional ability (assessed by tests such as sit to stand, timed up and go, 4 m walking test etc.) [1].The presence of the above accurate and reliable measurement tools to detect sarcopenia is not always available and applicable in clinical settings, so the evaluation and definition of sarcopenia seems to be time and cost consuming. Therefore, various international groups have published different diagnostic criteria for sarcopenia to enable health professionals in the early detection of this disorder. In 2010, the European Working Group (EWGSOP1) published its consensus criteria on sarcopenia in older people, which described low muscle mass and impaired muscle function as the primary parameters of the syndrome [1]. In its revised published definition, the European Working Group (EWGASOP2) stated low muscle strength cuff off points were also recommended as a primary parameter of sarcopenia, being a reliable and valuable measurement of muscle function [6,7].

Sarcopenia is well reported in literature to have a great impact upon quality of life and mortality. It appears to be also related to a high number of falls and hospital admissions due to fall injuries, with great socioeconomic costs. Thus, the early detection of sarcopenia is essential because it secures the initiation of effective treatment in a timely manner [8,9].

There is a variety of diagnostic screening methods, clinical tests, and assessment tools available for the clinical determination of sarcopenia in clinical practice. The clinical screening tools can be divided into self-reported questionnaires, anthropometric measurements, and a combination of the two above with physical functional tests [10]. The only available screening tools to evaluate sarcopenia in Greek are SarQol, which focuses on the assessment of life quality rather than the assessment of sarcopenia risk, and the SARC-F questionnaire, which is suggested as a primary screening tool by the EWGSOP2 algorithm to identify persons at risk for sarcopenia [11]. The latter is a brief self-reported questionnaire developed in English by Malstrom and Morley, translated and cross-culturally adapted into many languages, appearing to have good internal consistency, reliability and validity [12,13]. SARC-F was cross-culturally translated and validated into the Greek language and setting by Tsekoura et al. [14]. Based on the main features defining sarcopenia, the questionnaire consists of five domains that assess muscle strength, assistance in walking, rising from chairs, climbing stairs, and falls. However, the SARC-F questionnaire, as supported in the literature, is revealed to have a high specificity but low sensitivity, according to the EWGSOP2 criteria, therefore Rossi et al. designed an additional questionnaire (Mini Sarcopenia Risk Assessment questionnaire) as a pre-screening tool for sarcopenia risk assessment in the elderly, which is supported to be reliable and validated [15]. The SARC-F questionnaire has been referred to as being characterized mostly as a subjective evaluation that is performed by a subject or caregiver and is orientated towards assessing physical function and disability, while the MSRA questionnaire provides more objective and measurable answers in seven domains aimed at assessing muscle mass and strength loss [16,17].

Although the MSRA as a pre-screening tool for sarcopenia was elaborated to be used in Italian subjects, it has been validated in the Brazilian Portuguese, Polish, Chinese, and Thai languages as well [18,19,20,21]. Thus, the MSRA questionnaire shows a high sensitivity as a screening tool compared with the SARC-F for sarcopenia risk assessment, its use being essential to detect early sarcopenia, which can lead to a diagnosis. The aim of this study is to translate both versions of the MSRA questionnaire into the Greek language (Gr-MSRA), and validate its use in older subjects as an additional, low-cost, and easy pre-screening tool for sarcopenia, due to the remarkable prevalence reported in the older Greek population.

## 2. Materials and Methods

### 2.1. Mini Sarcopenia Risk Assessment Questionnaire (MSRA)

The tool of this survey is the Mini Sarcopenia Risk Assessment questionnaire (MSRA). This questionnaire consists of seven items (MSRA-7) in the full form and five items (MSRA-5) in the short form. Its design was based on the review of the literature regarding the risk factors of muscle mass and strength loss, two of the main characteristics of sarcopenia. It is composed of seven domains, which include the general assessment of the subjects; therefore, there are three questions regarding age, physical activity level and the number of hospitalizations throughout a year, one question about weight loss and three more questions concerning dietary assessment (number of meals per day, dairy product consumption, protein consumption). In the MSRA-5 version, two of the dietary questions (dairy product and protein consumption) are excluded [15,16].

The scores given to the items in the MSRA-7 are 0, 5 and 10 points. The total score in the full form can be a minimum of 0 and a maximum of 40 points, with a value of 30 points or lower to be indicative of sarcopenia. In terms of the MSRA-5 version, the scores given are 0, 5, 10 or 15 points. The total score for the short form can be a minimum of 0 and a maximum of 60 points, with a value of 45 points or lower to indicate sarcopenia [15] (Table S1 (English version), Table S2 (Greek version)).

### 2.2. Study Design and Participants

The study was conducted from April 2021 to June 2022 and the participants were recruited at the Internal Medicine and Orthopaedic outpatient departments of the General Hospital of Heraklion, “Venizeleio-Pananeio”. Ninety participants were enrolled in the main study. Inclusion criteria were as follows: (a) at least 65–89 years old; (b) had normal cognitive function; (c) were able to walk; (d) had no mobility impairment. For the collection of the data, the sampling method of convenience was used. Participants completed demographic questions regarding age (years) and biological gender (male/female).

The study was approved by the scientific research committee of the hospital. All the participants signed an informed consent for this research. Before proceeding to the translation and cross-cultural adaptation, authorization from Dr A. Rossi, co-author of the original MSRA, was taken and consent for the Greek versions of the MSRA-7 and MSRA-5 was given via e-mail. 

### 2.3. Translation and Cultural Adaptation Phase

The translation and cross-cultural adaptation of the two versions of the MSRA questionnaire was undertaken according to the methodology guidelines for the translation and intercultural adaptation of health questionnaires, as provided by the WHO [22].

The MSRA questionnaire was translated and culturally adapted into the Greek language according to the “Minimal Translation Criteria” [23]. In the first phase of this process, a standardized translating forward procedure was used to develop a Greek version of both MSRA questionnaires. Two different people proceeded in the independent translation of the original forward translated version. After this phase, the two translated adaptations were assessed by a third person, who was able to obtain an agreed translation (reconciliation version). The agreed version was then translated into the language of the original questionnaire (backward translation) by a bilingual person who was a professional translator, but without knowing the standard form of the questionnaire.

The translated questionnaire was then completed by 10 persons from the target population to assess the apparent validity (Face Validity). In the next phase, the questionnaire was assessed by 3 experts in the field of sarcopenia, who had the cognitive ability to evaluate the tool and to propose possible approaches for improving it (Content Validity). Items with a Coefficient Validity Ratio (CVR) > 0.70 were preserved in the final version of the instrument. The Content Validity Index (CVI) for the instrument, considering a value >0.80 to be adequate, was also calculated [24].

The multidisciplinary committee gave permission to continue the validation process of the Greek versions of the MSRA, so a pilot study was performed to test the cultural adaptation, in 10 subjects (both female and male) initially, with good mental function. Then, the intra-rater reliability (test-retest) of both the MSRA-7 and MSRA-5 was assessed by the same researcher, with a time interval of two weeks, in 16 different subjects.

### 2.4. Validity Phase

The validation analyses in this study relied on the correlation of the Greek versions of the MSRA-7 and MSRA-5 with the SARC-F questionnaire, which is translated and validated in Greek and has been proven to be a reliable pre-screening tool for sarcopenia in the elderly [13] (Table S3).

### 2.5. Statistical Analysis

The intra-rater reliability was determined by calculating the Intra-class Correlation Coefficient (ICC), on the assessment of the data initially and after a 2-week interval. Values below 0.5 indicate poor reliability, between 0.5 and 0.75 indicate moderate reliability, between 0.75 and 0.9 indicate good reliability, and any value above 0.9 shows excellent reliability. The minimum sample size was estimated to be 16 people after setting for a minimum acceptable reliability of 0.75, an expected reliability of 0.95, a significance level of 5%, a power of 99%, and an expected abandonment rate of 10% [25].

The concurrent validity between the MSRA questionnaire and the SARC-F questionnaire was assessed using the Spearman’s rank correlation coefficient (ρ). Correlation coefficients of ρ < 0.30 were considered as negligible; 0.30–0.50 as low; 0.50–0.70 as moderate; 0.70–0.90 as high; and ρ > 0.90 as very high [26]. The determination of the sample size for the Spearman’s rank correlation coefficient was based on 2-tailed α ≤ 0.01, statistical power greater than 99%, and a correlation threshold value for the correlation coefficient of 0.50. Based on these assumptions, the minimum sample size required was n = 83 [27].

The statistical analyses were conducted using IBM SPSS (version 25). For all tests, the statistical differences were determined to be significant at *p* < 0.05.

### 2.6. Ethical Considerations

This study was conducted in full compliance with the new General Data Protection Regulation (GDPR) [EU 2016/679] 25.5.2018 concerning sensitive personal data, and all relevant licenses were secured prior to data collection. Therefore, this study was approved by the Department of Nursing, School of Health Sciences, Hellenic Mediterranean University (No ref. 4638. 29.3.2021) and by the Scientific Research Committee of the Hospital for the data being collected (Ref No. 44-5/22-4-2021). The participants were informed that the procedure was anonymous and that their personal data and answers would be used exclusively for research purposes; they all consented to fill in the questionnaires.

## 3. Results

The total number of participants was 90, of which 56 (62.2%) were females and 34 (37.8%) males, aged between 65 and 89 years (M = 74.9, SD = 6.5). Of all the participants, a random sample of 16 people participated in the intra-rater reliability analysis. The sample comprised of nine (56.3%) females and seven (43.7%) males, aged between 65 and 89 years (M = 75.8, SD = 6.0).

### 3.1. Translation and Adaptation

Concerning the questionnaires’ comprehension and cultural diet relevance, one modification was required. Thus, the term “ragout”, which is not well known in Crete, was replaced by another local term relevant to the Mediterranean diet and with similar meaning (in terms of the ingredients and way of cooking). The multidisciplinary team approved the development of the final versions of the MSRA and Dr A. Rossi also gave his consent. The apparent validity was assessed in 10 participants, to confirm that the scale contained items consistent with the attribute measured, thus does not lead to incomplete or misleading answers to questions.

### 3.2. Validity

The assessment of the scores of the MSRA-7 and MSRA-5 questionnaires showed that they correlated with those of the SARC-F questionnaire in both Greek versions, as described in the Section 2, with the CVR results showing that 100% of the items (n = 7) were acceptable.

The ICC of the Greek MSRA questionnaire between the initial assessment and reassessment was 0.986 (CI 95%; 0.961–0.995). The scores had an excellent consistency between the two occasions as indicated by the estimated coefficient.

The Greek MSRA-5 questionnaire had a very high correlation with the SARC-F questionnaire (rho) = −0.724, *p* < 0.001). In addition, the Greek MSRA-7 questionnaire had a very high correlation with the SARC-F questionnaire (rho = −0.741, *p* < 0.001).

## 4. Discussion

Considering the progress of the aging population globally, and with all of the socioeconomic consequences, the early diagnosis of sarcopenia is crucial in any public health service. Sarcopenia syndrome appears with a different prevalence in nursing homes, in hospitalized patients, in community dwelling adults and in different cultures with variable lifestyles. Therefore, it is quite difficult to estimate its prevalence objectively with one common easy assessment tool. However, prioritizing the early detection of sarcopenia, which is related to frailty, muscle atrophy, reduced muscle strength and balance, could possibly prevent, or decrease, functional impairment via the application of appropriate prevention or therapeutic interventions. It seems that even identifying the risk factors of probable sarcopenia is essential, to initiate early treatment to prevent sarcopenia. According to EWSOP2, probable sarcopenia is the basis on which interventions must be initiated. Therefore, pre-screening questionnaires that gauge the risk of sarcopenia, and which can be easily performed in clinical practice, are an essential tool for the detection and early diagnosis of this condition [28]. Although there are a variety of screening tools used to detect sarcopenia, according to the EWSOP2 criteria, SARC-F is one of the best screening tools in clinical practice, that can be translated, adapted, and validated in many languages [29,30,31]. SARC-F is a well-known and accepted pre-screening tool used in research and in clinical practice; however, its sensitivity is low, and its specificity is high [32]. Based on the above facts and on the EWGSOP2 criteria, Rossi et al. [15] designed and validated a new pre-screening tool with seven domains, including questions about dietary habits (dairy product and protein consumption), with consideration to how malnutrition in older people has been evaluated and dietary elements, such as proteins and caloric intake, have been identified that are related to muscle mass and strength loss [33].

The MSRA questionnaire, which is a quick self-reported questionnaire used to evaluate, primarily, the risk of sarcopenia in older people, has been translated into and validated in four languages. Many studies have been conducted to assess the reliability and statistical strength of the MSRA questionnaire, which shows high sensitivity compared to the SARC-F and is suggested for use as a pre-screening tool. To the best of our knowledge, this is the first study to translate, adapt and validate both versions of the MSRA questionnaire into the Greek language. The translation and validation procedures followed in this study were performed according to established guidelines. The process of the translation presented with no major problems, with only one adjustment needed to be performed in the dietary domains, due to different cultural habits, to make the questionnaire more comprehensive in the Greek population. The back-translation was sent to Dr A. Rossi, who gave his consent for the Greek version, enabling us to proceed with the validation process. The MSRA questionnaires were validated by comparing them with the SARC-F questionnaire, which is recommended as a reliable and validated pre-screening tool by the EWGSOP2 for the detection of sarcopenia in the clinical population [5,9,11]. The SARC-F has been translated, adapted, and validated in the Greek population by Tsekoura et al., hence why it was selected to be correlated with the MSRA versions [14]. The MSRA-5 questionnaire in Greek showed a very high correlation with the SARC-F questionnaire (rho = −0.724, *p* < 0.001) and the MSRA-7 questionnaire had a very high correlation with the SARC-F questionnaire (rho = −0.741, *p* < 0.001). Therefore, the Greek versions of the MSRA-7 and MSRA-5 may also be considered to be reliable and validated easy to use tools for the detection of sarcopenia in the Greek population.

The MSRA questionnaire, elaborated in Italian subjects, presented high sensitivity compared to the SARC-F, making it a suitable pre-screening tool for sarcopenia in community dwelling healthy older subjects. It has been highlighted that the questionnaire can reveal the special characteristics of sarcopenia [15]. The MSRA questionnaire, in the Brazilian-Portuguese translated version, presented high reliability and agreement between the seven and five items, in hospitalized cancer patients. It was suggested as an easy nutritional assessment tool to detect early sarcopenia in patients with lymphomas, leukemias and intestinal tumors [18]. The MSRA questionnaire in the Chinese language has been validated and it was concluded that the C-MSRA-5, compared with the C-MSRA-7, is a better tool for screening sarcopenia in community dwelling older adults [19]. The Polish versions of the MSRA presented good sensitivity, with better reliability of the MSRA-5 than the MSRA-7 when used as a screening tool for sarcopenia in community dwelling adults [20]. The Thai version of the MSRA-7 questionnaire provided more sensitivity with limited specificity than the MSRA-5 for screening sarcopenia in older patients at a medical outpatient clinic [21].

By evaluating all of the studies conducted to translate, adapt and validate the MSRA questionnaire in the languages referred to above, it can be concluded that although there are differences in the research methodology, subject sample chosen and different sarcopenia prevalence, it seems to be a reliable pre-screening tool for sarcopenia. The Greek versions of the MSRA questionnaire (Gr-MSRA) also presented significant validity and will be useful as pre-screening tools for the early diagnosis of sarcopenia, due to the significant prevalence of this muscle disorder in the older Greek population.

### Limitations

Some limitations of our study are: (a) the sample consisted of older Greek volunteers visiting the outpatient’s department at a hospital setting, (b) the sensitivity and specificity of the Greek versions were not assessed due to the methodology chosen to validate the tool. However, the average age of the sample was 74.9, which is expected to have a higher prevalence of sarcopenia.

As only a few validation analyses were conducted for the MSRA questionnaires, in different settings and methodologies, more studies should be completed in future to correlate this screening tool with other suggested pre-screening questionnaires given in the literature. Furthermore, studies with a larger sample size and various clinical settings will define the usefulness of the MSRA as a low-cost and reliable tool to facilitate the early screening of sarcopenia.

## 5. Conclusions

The MSRA-5 and MSRA-7 versions of the MSRA questionnaire were cross-culturally adapted and validated in a clinical setting for use as a pre-screening tool for sarcopenia in older Greek adults. The Greek versions of the MSRA showed acceptable reliability measures and significant correlation with the Greek version of the SARC-F questionnaire, therefore they can be used in the Greek population, in clinical practice, or in research, as a pre-screening tool for sarcopenia. Since both the MSRA and SARC-F questionnaires are indicative tools for sarcopenia, further testing should also be undertaken in older Greek adults, to finally confirm a diagnosis of sarcopenia.

## Data Availability

Data supporting the reported results in this study are available on request from the corresponding author. The data are not publicly available due to ethical restrictions.

## Supplementary Materials

The following supporting information can be downloaded at: https://www.mdpi.com/article/10.3390/nursrep13010037/s1, Table S1: MSRA-7 and MSRA-5 questionnaires; Table S2: Σύντομη εκτίμηση επικινδυνότητας της Σαρκοπενίας (7 και 5 ερωτήσεων); Table S3: SARC-F questionnaire.
