# Supplementary material for: Greek Translation, Cultural Adaptation and Validation of the Mini Sarcopenia Risk Assessment Questionnaire, to Evaluate Sarcopenia in Greek Elderly at a Hospital Setting
Source: Nurs Rep. 2023 Mar 3;13(1):404–11. doi: 10.3390/nursrep13010037 (PMC10058970; doi:10.3390/nursrep13010037)
Supplement: Supplementary file 1 [file nursrep-13-00037-s001.zip › nursrep-2157270-supplementary.pdf]

Table S1. MSRA-7 and MSRA-5 questionnaires.

	7 items	5 items
1-How old are you?	Score	Score
≥70 years	0	0
<70 years	5	5
2-Were you hospitalized in the last year?		
Yes, and more than one hospitalization	0	0
Yes, one hospitalization	5	10
No	10	15
3-What is your activity level?		
I'm able to walk less than 1000 meters	0	0
I'm able to walk more than 1000 meters	5	15
4-Do you eat 3 meals per day regularly?		
No, up to twice per week I skip a meal (for example I skip breakfast or I have only milky coffee or soup for dinner)	0	0
Yes	5	15
5-Do you consume any of the following?		
Milk or dairy products (yogurt, cheese), but not every day	0	-
Milk or dairy products (yogurt, cheese) at least once per day	5	-
6- Do you consume any of the following?		
Poultry, meat, fish, eggs, legumes, ragout or ham, but not every day	0	-
Poultry, meat, fish, eggs, legumes, ragout or ham at least once per day	5	-
7-Did you lose weight in the last year?		
>2 kg	0	0
≤2 kg	5	10

Adopted from Rossi et al. [1].

Table S2. Σύντομη εκτίμηση επικινδυνότητας της Σαρκοπενίας (7 και 5 ερωτήσεων).

	7 ερωτήσεις	5 ερωτήσεις
	**Σκορ**	**Σκορ**
**1. Πόσο χρονών είστε;**		
≥ 70 ετών	0	0
< 70 ετών	5	5
**2. Νοσηλευτήκατε τον περασμένο χρόνο;**		
- Ναι, και περισσότερες από μία νοσηλεία	0	0
- Ναι, μία νοσηλεία	5	10

- Όχι	10	15
**3. Ποιο είναι το επίπεδο δραστηριότητάς σας;**		
- Μπορώ να περπατήσω λιγότερο από 1000 μέτρα	0	0
- Μπορώ να περπατήσω πάνω από 1000 μέτρα	5	15
**4. Τρώτε τακτικά 3 γεύματα την ημέρα;**		
- Όχι, έως και δύο φορές την εβδομάδα παραλείπω ένα γεύμα (για παράδειγμα παραλείπω το πρωινό ή έχω μόνο καφέ με γάλα ή σούπα για δείπνο)	0	0
- Ναι	5	15
**5. Καταναλώνετε κάποιο από τα παρακάτω;**		
- Γάλα ή γαλακτοκομικά προϊόντα (π.χ. γιαούρτι, τυρί), αλλά όχι κάθε μέρα	0	-
- Γάλα ή γαλακτοκομικά προϊόντα (π.χ. γιαούρτι, τυρί) τουλάχιστον μία φορά την ημέρα	15	-
**6. Καταναλώνετε οτιδήποτε από τα παρακάτω;**		
- Πουλερικά, κρέας, ψάρι, αυγά, όσπρια, κοκκινιστά ή ζαμπόν, αλλά όχι κάθε μέρα	0	-
- Πουλερικά, κρέας, ψάρι, αυγά, όσπρια, κοκκινιστά ή ζαμπόν τουλάχιστον μία φορά την ημέρα	5	-
**7. Χάσατε βάρος το προηγούμενο έτος;**		
> 2 κιλά	0	0
≤ 2 κιλά	5	10

Table S3. *SARC-F questionnaire.*

Component	Question	Scoring
Strength	How much difficulty do you have in lifting and carrying 10 pounds?	None= 0<br>Some=1<br>A lot or unable=2
Assistance in walking	How much difficulty do you have walking across a room?	None= 0<br>Some=1<br>A lot, use aids or unable=2
Rise from a chair	How much difficulty do you have transferring from a chair to bed?	None= 0<br>Some=1<br>A lot or unable without help=2
Climb stairs	How much difficulty do you have climbing a flight of 10 stairs?	None= 0<br>Some=1<br>A lot or unable=2
Falls	How many times have you fallen in the past year?	None= 0<br>1-3 falls=1<br>4 or more falls=2

Reprinted/adapted from Malmstrom et Morley [2]

Reference

1. Rossi, A.P.; Micciolo, R.; Rubele, S.; Fantin, F.; Caliari, C.; Zoico, E.; Mazzali, G.; Ferrari, E.; Volpato, S.; Zamboni, M. Assessing the risk of sarcopenia in the elderly: The Mini Sarcopenia Risk Assessment (MSRA) Questionnaire. *J. Nutr. Health Aging* **2017**, *21*, 743–749. https://doi.org/10.1007/S12603-017-0921-4.
2. Malmstrom, T.K.; Morley, J.E. SARC-F: A simple questionnaire to rapidly diagnose sarcopenia. *J. Am. Med. Dir. Assoc.* **2013**, *14*, 531–532. https://doi.org/10.1016/j.jamda.2013.05.018.
